# Nitrogen oxide reduction through absorbent solutions containing nitric acid and hydrogen peroxide in hollow fiber membrane modules

**DOI:** 10.1016/j.heliyon.2019.e02987

**Published:** 2019-12-06

**Authors:** Sutrasno Kartohardjono, Clarissa Merry, Mohamad Sofwan Rizky, Catharina Candra Pratita

**Affiliations:** Department of Chemical Engineering, Faculty of Engineering, Universitas Indonesia, Indonesia

**Keywords:** Chemical engineering, Environmental chemical engineering, Chemical reaction engineering, Hydrogen peroxide, Nitric acid, NOx, Reduction efficiency, Wet method

## Abstract

Emissions of nitrogen oxides such as NO and NO_2_, which are commonly known as NOx, are threats to human existence and cause environmental problems. Mainly, two techniques have been developed to drastically reduce these emissions, which are dry and wet processes. The wet process has several advantages, major identifiable advantages are the adaptability to the flue gas, low operating temperatures and no poisoning and inactivation catalyst. Also, a mixture of hydrogen peroxide and nitric acid are used as absorbents solution for NOx reduction in the wet process. The advantages of using this mixture include the ability to reduce the negative effect of NOx and does not contaminate the scrubbing solution. In addition, nitric acid has an economical advantage in the process considering the fact that it is produced in the process. Finally, it can be conducted at ambient temperature. This study furthermore used a mixture of hydrogen peroxide and nitric acid solutions as an absorbent to reduce NOx in hollow fiber membrane modules. The hydrogen peroxide oxidized HNO_2_ to nitric acid, while enhances the oxidation through an autocatalytic reaction. The effects of the feed gas flow rate, hydrogen peroxide concentrations and number of fibers on the NOx reduction, absorbed NOx and flux were varied to study. The experimental results showed that the increase in the feed gas flow rate from 100 to 200 mL/min decreased NOx reduction from about 98 to 94% but increased the absorbed NOx and flux from about 0.13 to 0.255 mmol/h and 0.85–1.63 mmol/m^2^.h, respectively The increase in proportion of NO_x_ in the feed gas effect was dominant than the increase in absorbed NOx. An increase in hydrogen peroxide concentration from 0.5 to 10 wt.% in the absorbent solutions increased NOx reduction, absorbed NOx and flux from about 94 to 98%, 0.257–0.267 mmol/h and 1.09–1.13 mmol/m^2^.h, respectively. Additionally, the H_2_O_2_ plays an important role in enhancing HNO_2_ oxidation to HNO_3_. Furthermore, an increase in the number of fibers from 50 to 150 in the membrane module increased NOx reduction and absorbed NOx from 86 to 97% and 0.23–0.27 mmol/h. Flux decreased from 2.98 to 1.13 mmol/m^2^.h due to increment in the gas-liquid contact surface area.

## Teaser

Chemical engineering; Environmental chemical engineering; Chemical reaction engineering; Environmental chemical engineering; Hydrogen peroxide; Nitric acid; NOx; Reduction efficiency; Wet method.

## 1 Introduction

Nitrogen oxides, such as NO and NO_2,_ commonly known as NOx are usually emitted from the consumption of fossil fuel in power generation and industrial production, endanger life and pose a threat to the environment [1, 2]. NO_*x*_ has been considered one of the major air pollutants, which do not only harm the human body, but also trigger a series of serious environmental problems like global warming, photochemical smog, ozone depletion, and respiratory diseases [3, 4, 5]. NOx further reacts with OH^−^ radicals in the atmosphere to form nitric acid, thereby producing acid rain [6]. In addition, NOx causes the eutrophication of lakes, which brings to extinction of aquatic life [7]. Scientists and engineers all over the world have been challenged by this fact, devising ways of developing a more efficient deNOx technique, aimed at mitigating the effect of strict NOx emission [8, 9].

The emissions of NO_x_ mainly depend on the combustion temperature, time, and air-fuel ratio [10]. Several technological techniques have been developed to reduce NO_x_ emissions in order to meet regulations and mainly based on two methods: dry and wet processes [11]. The dry process includes selective catalytic reduction (SCR) [12] and NO_x_ storage and reduction (NSR) [13], which is also known as lean NO_x_ trap (LNT) [8]. SCR using NH_3_ has the reducing reagent over catalysts based on V_2_O_5_-WO_3_/TiO_2_ or Cu- and Fe-zeolites [14, 15]. This has been proven highly efficient for NO_x_ removal, involving flue gas temperatures typically ranging from 300 to 400 °C [16]. The commercial V_2_O_5_-WO_3_/TiO_2_ catalyst shows high NO_x_ removal efficiency (>90%) at 350–400 °C [17]. However, it is not suitable for medium and small industry boilers due to its high cost, its limitation to low-temperature for SCR application [18, 19], and the introduction of ammonia into the gas stream thereby leading to the formation of ammonium bisulfate and ammonia slip in the atmosphere [20]. The deactivation of SCR catalyst due to the high concentration of fly ash also makes this technology unreliable for prolonged efficient operation [21, 22]. The method, adopted by NSR, is being influenced by sulfur poisoning or thermal reduction of the catalyst being used in the process, which also aids production of side products such as N_2_O and NH_3_, identified as strong greenhouse gas and toxic materials.

The wet process offer several advantages over the dry these include the adaptability to the flue gas, the low operating temperature and no catalyst poisoning and inactivation or decrease ability as time goes [23]. To neutralize NOx or convert the insoluble NOx species by oxidation to more soluble ones, the wet process uses various absorbents such as aqueous solutions of sodium or calcium hydroxide, hydrogen peroxide, sodium chlorine or potassium permanganate, added to the scrubbing solutions. The use of hydrogen peroxide as the oxidizing agent are considered appropriate due to the fact that it has the capacity to reduce the gaseous pollutants without producing liquid wastes. Then, the product from the oxidation could be recovered and recycled [11].

In this study, hollow fiber membrane modules was utilized for NO_x_ reduction into absorbent solutions containing hydrogen peroxide and nitric acid as oxidizing and autocatalytic agents, respectively. The hydrogen peroxide is an oxidizing agent, widely used in the chemical industry such as bleach for textiles and pulp, and a treatment for municipal and industrial waste. The use of hydrogen peroxide and nitric acid to scrub NOx is an attractive choice considering the fact that it could handle a wide rates of NOx. It does not produce contaminants to the scrubbing solutions and gives the products in commercial quantity from the process, i.e. nitric acid [20]. In addition, the process could be conducted at ambient temperature [24] and provide high NOx reduction [25].

Gas absorption using membrane modules is a combined process that fully integrates the advantages of membrane separation and absorption processes. In the hollow fiber membrane module, there are two different spaces for each fluid: the shell side and the lumen fiber. The absorbent solutions located in the shell side of the hollow fiber membrane module, has a selective absorption for several types of gas species. Also, the pores in the membrane fiber play a role in distributing feed gas into the shell side of the membrane module containing nitric acid and hydrogen peroxide, thereby enlarging the contact area between the gas and liquid phases where the reaction between the NOx and absorbent solution takes place. In addition, hollow fiber membrane module offers operational flexibility to scale up and down [26, 27].

The mechanism of NOx absorption into solutions containing H_2_O_2_ and HNO_3_ has been described in several sources [24, 28, 29]. The gas-phase reactions that occur when NO and NO_2_ are mixed in the presence of O_2_ and water vapor are as follow:

(1) 2NO + O2 → 2NO_2_

(2) NO + NO_2_ ↔ N_2_O_3_

(3) 2NO_2_ ↔ N_2_O_4_

(4) NO + NO_2_ + H_2_O ↔ 2HNO_2_

Furthermore, in the liquid phase there are transport of NO, HNO2, NO2, N2O3 and N2O4 compounds through the interface to the bulk liquid, where the last 3 species react with water to form nitrous and nitric acids according to:

(5) 2NO_2_ + H_2_O → HNO_2_ + HNO_3_

(6) N_2_O_3_ + H_2_O → 2HNO_2_

(7) N_2_O_4_ + H_2_O → HNO_2_ + HNO_3_

The presence of hydrogen peroxide in the liquid phase prevents the decomposition of HNO_2_ by oxidation to HNO_3_ through the following reaction [24]:

(8) HNO_2_ + H_2_O_2_ ↔ HNO_3_ + H_2_O

Furthermore, the addition of nitric acid solution enhances the rate of reaction Eq. (8) via an autocatalytic reaction [28].

## 2 Materials and methods

### 2.1 Experimental setup

**Figure 1:**
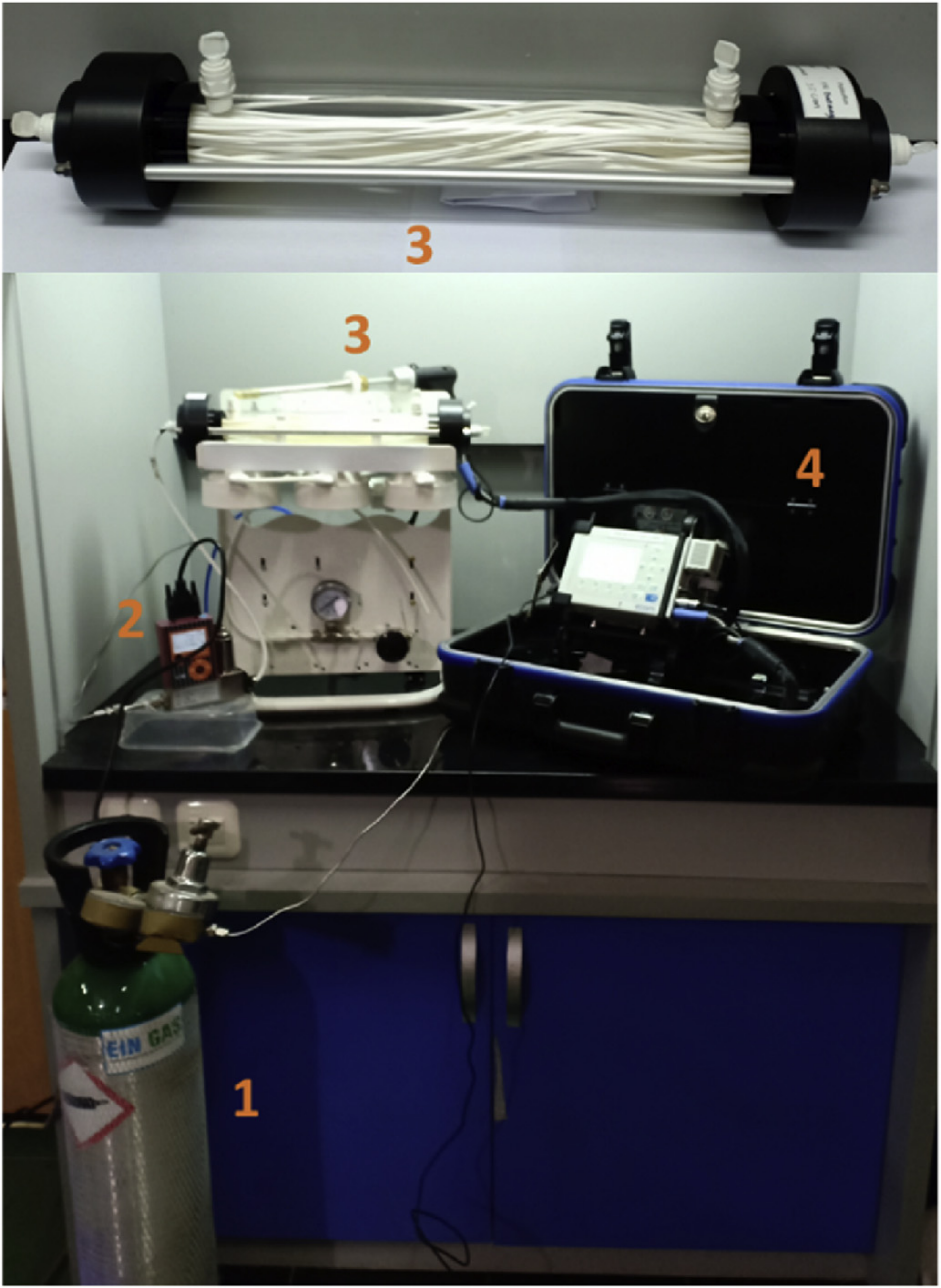
Schematic diagram of experimental set up: 1. Feed gas containing NOx tank; 2. Mass flow controller; 3. Hollow fiber membrane module; 4. Gas analyzer.

There are two channels in the hollow fiber membrane module in which lumen bundle and shell side of the module for inlet and outlet fluids. For experimental purposes, one channel in both the lumen bundle and shell side of the module was plugged thereby allowing only one channel each in both parts. A certain volume of the absorbent solution, composed of H2O2 and HNO3, was statically placed in the shell side, while the feed gas containing NOx flowed through the lumen fiber and penetrated into the fiber through the shell side of the membrane module. This was easily achieved considering the fact that there was a contact between the feed gas and absorbent solution. The feed gas flow rates to the membrane module was controlled with a mass transfer controller. This makes the membrane fibers in the contactor distributes the feed gas before it contacts the liquid in the shell side in order to increase the surface area for the gas–liquid contact. The hollow fiber module details and the operating conditions are summarized in Tables 1 and 2, respectively. Meanwhile, the schematic diagram of experimental set up is presented in Figure 1.

**Table 1:** Hollow fiber membrane module dimensions.

Hollow fiber membrane module	Sizes
Shell diameter	2.5 and 5 cm
Module length	25 cm
Number of fibers	32, 50, 100, and 150

**Table 2:** Operating conditions.

Conditions	Values
Feed gas flow rates	100, 150, and 200 cm^3^/min
Feed [NOx] ppm	560
[HNO_3_] M	0.5 ± 0.02
[H_2_O_2_] wt.% Temperature	0.25, 0.5, 1, 5 and 10 ± 2% 27 ± 3 °C

### 2.2 Materials and analytical methods

Four hollow fiber modules used were supplied by GDP Filter Bandung, in Indonesia. These modules consisted of 32, 50, 100, and 150 fibers, made up of polysulfone; having inner and outer diameters of 0.18 cm and 0.2 cm respectively. H2O2 (35 wt.%) and HNO3 (65 wt.%) were purchased from Merck, Indonesia, while the feed gas containing approximately 560 ppm of NOx in air was supplied by Energi Indogas Nusantara, Indonesia. During the experiment, the feed gas flow rates were controlled using a CX Series mass flow controller from Shanghai Cixi Instrument. The NOx concentrations in the inlet and outlet of the membrane modules were measured using combustion gas analyzer Ecom-D. To prepare 1 L 0.5 M HNO3, 35 mL of HNO3 (65 wt.%) was added to 965 mL distilled water in a volumetric flask. This solution was then titrated with sodium hydroxide to determine its concentration. Moreover, 7.5, 15, 30, 150, and 300 ml of H2O2 (30 wt.%) were diluted with distilled water up to 1000 mL to prepare 1 L of 0.25, 0.5, 1, 5 and 10 wt.% H2O2, respectively. These solutions were quantitatively analyzed by the permanganometry method through the addition of sulfuric acid and titration with the potassium permanganate. In the module containing 32 fibers, 25 ml of 0.25, 0.5, 1, 5 and 10 wt.% of H2O2 and 25 ml of HNO3 0.5 M were added to the contactor in the shell side. Meanwhile, in the module containing 50, 100 and 150 fibers 75 ml of 0.5, 5 and 10 wt.% of H2O2 and 75 ml of HNO3 0.5 M were added to the contactor in the shell side.

### 2.3 Experimental parameters

The amount of Absorbed NOx by the absorbent solution, *NOx*_Abs_, flux, *J* and NOx reduction, *R*, during the experiments were calculated as follows [25, 30]:

(9) $$NOx_{Abs}=\left(NOx_{in}-NOx_{out}\right)Q_{G}\frac{P}{RT}$$

(10) $$J=\frac{NOx_{Abs}}{A_{m}}$$

(11) $$R=\;\frac{NOx_{in}-NOx_{out}}{NOx_{in}}$$

where *NOx*_in_ and *NOx*_out_ are absorbed NOx concentrations in the feed gas entering in and leaving the membrane contactor, respectively. In addition, *Q*_G_, *P*, *T*, *R*, and *A*_m_ are the feed gas flow rate, atmospheric pressure, temperature, ideal gas constant, and membrane fiber surface area, respectively.

## 3 Results and discussion

### 3.1 Influence of feed gas flow rates

The mechanism of NOx absorption in the absorbent solution followed three steps of transfer [25, 31]: The transfer of NOx from the bulk gas to the inside of the lumen fiber in the gas phase, transfer of NOx through the pores of the membrane fibers to the outside of the fiber in the gas phase; and transfer of NOx to the bulk absorbent solutions. The reactions occur between NOx in the gas stream and the absorbent solution as in Eqs. (5), (6), (7), and (8) when the gas stream containing NOx is in contact with the absorbent solution in the gas-liquid interface and in the bulk of the absorbent solutions at the shell side of the membrane module.

**Figure 2:**
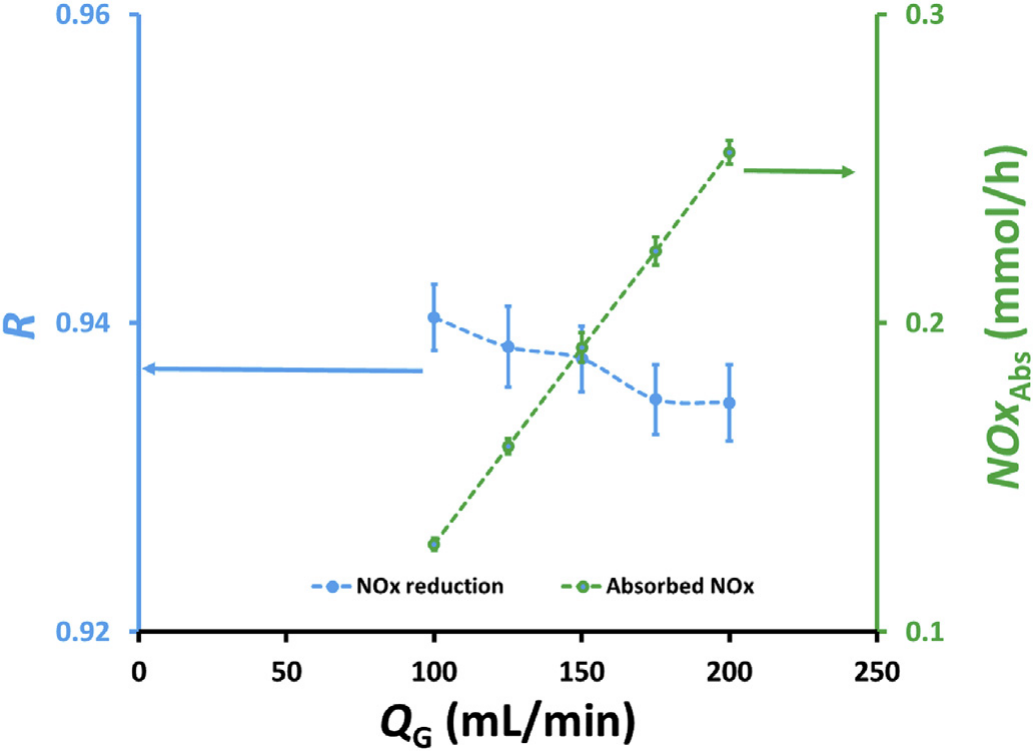
Effects of feed gas flow rate, *Q*_G_, on NOx reduction, *R*, and amount of absorbed NOx by the absorbent solution, *NOx*_Abs_, containing a mixture of 25 mL of 0.5 wt.% H_2_O_2_ and 25 mL 0.5 M HNO_3_ in a hollow fiber membrane module with 32 fibers.

Figure 2 shows the effects of feed gas flow rate on NOx reduction, *R*, and absorbed NOx, *NOx*_Abs_, in a hollow fiber module containing 32 fibers. As shown in Figure 2, the absorbed NOx increased with increasing gas flow rate, but the NOx reduction only slightly decreased. Increasing the feed gas flow rate will increase the amount of NOx in the feed gas, which is distributed in the lumen fibers, thereby increasing the number of moles of NOx gas, which spreads out from the lumen fibers to the pores of the membrane fibers toward the shell side containing the absorbent solutions where the reactions (Eqs. (5), (6), (7), and (8)) occurred. Therefore, increasing the feed gas flow rate increased absorbed NOx, which in turn enhanced the NOx reduction. Although the increase in the feed gas flow rate increased the amount of NOx in the feed gas, it was disadvantageous for the NOx reduction, as expressed in Eq. (11). The slightly decrease of NOx reduction as presented in Figure 2 indicated that the effect of the amount of NOx in the feed gas is almost similar to the effect of absorbed NOx. The NOx reduction slightly decreased from 0.940 to 0.935 when the feed gas flow rate increased from 100 to 200 mL/min, indicating that the increase in feed gas flow rate have no significant impact on the NOx reduction. Meanwhile, Wang and Yu [25] reported in their study, NO reduction efficiency decreased from approximately 0.91 to 0.29 when the feed gas flow rate increased from 50 to 250 mL/min by using NO concentration in the feed gas of ~184.8 ppm, absorbent solutions containing a mixture of 0.2 wt.% H2O2 and 5 wt.% NaCl, and polypropylene-based hollow fiber membrane module. This result confirms that the increase in the feed gas flow rate will increase absorbed NOx, but also increase the NOx concentration of the gas leaving the membrane module. The effect of feed gas flow rate on NOX reduction in this study is somewhat different from what reported by Wang and Yu [25]. In this study there was a direct contact between the absorbent solution in the shell side of membrane module and the feed gas that flew through the lumen fiber and diffused to the membrane pores to the shell side of the contactor, so that there was no significant effect on the NOx reduction with increasing feed gas flow rate. Meanwhile, the experiments conducted by Wang and Yu the gas-liquid contact only occurred in the pores of the membrane fibers so that there was a drastic decreased in NOx reduction with increasing feed gas flow rate.

**Figure 3:**
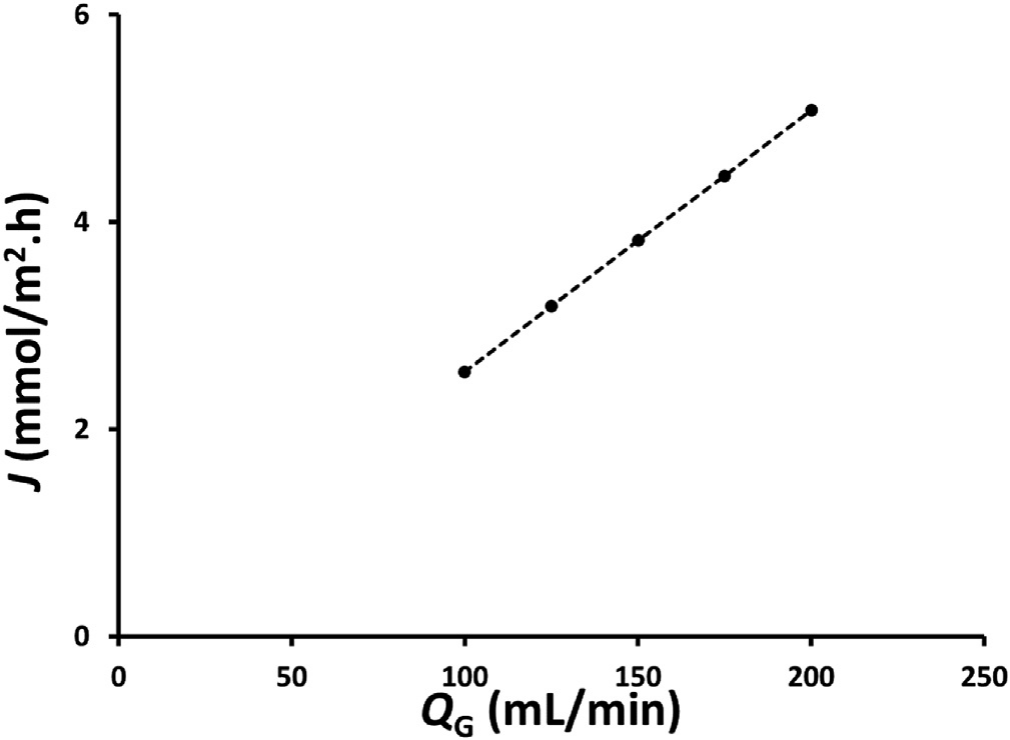
Effects of feed gas flow rate, *Q*_G_, on the flux, *J*, in a hollow fiber membrane module with 32 fibers containing a mixture of 25 mL of 0.5 wt.% H_2_O_2_ and 25 mL 0.5 M HNO_3_ as an absorbent solution.

The effects of the feed gas flow rate on the flux is shown in Figure 3. The flux increased with increasing feed gas flow rate. The increase in the feed gas flow rate not only increasing gas flow rate to the membrane module, *Q*_G_, but also increasing *NOx*_Abs_, as shown in Figure 2; thus, the same membrane surface area provides a higher flux, as shown in Figure 3. As can be seen from Figure 3, the flux increased from 2.6 to 5.1 mmol/m^2^.h, when the feed gas flow rate increased from 100 to 200 mL/min. Wang and Yu [25] reported that the NO absorption flux increased from ~0.025 to 0.040 mmol/m^2^.h when the feed gas flow rate increased from 50 to 200 mL/min by using NO concentrations in the feed gas of ~184.8 ppm, absorbent solutions containing a mixture of 0.2 wt.% H2O2 and 5 wt.% NaCl, and polypropylene-based hollow fiber membrane module. The absorption rate in this study was much higher than that obtained by Wang and Yu [25] because in this study there was a direct contact between feed gas containing NOx and the absorbent solution in the shell side of the membrane module, whilst the gas-liquid contact only occurred in the pores of membrane fiber in Wang and Yu experiments [25].

**Figure 4:**
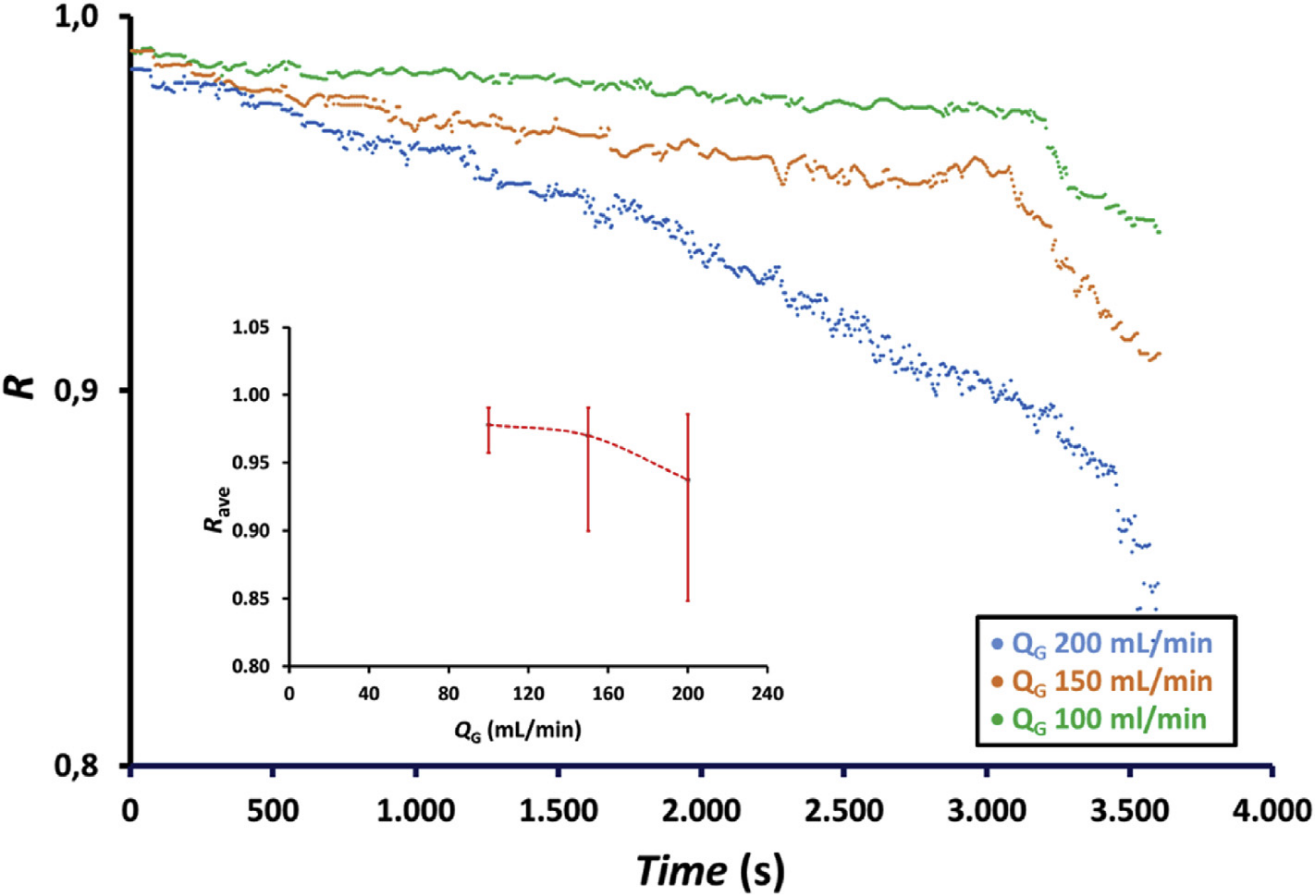
Profile of NOx reduction, *R*, with time at feed gas flow rates, *Q*_G_, of 100, 150, and 200 mL/min and the effect of feed gas flow rates on the average NOx reduction, *R*_ave_ (inset figure) using a membrane module with 100 fibers and an absorbent solution containing a mixture of 75 mL of 0.5 wt.% H_2_O_2_ and 75 mL of 0.5 M HNO_3_.

**Figure 5:**
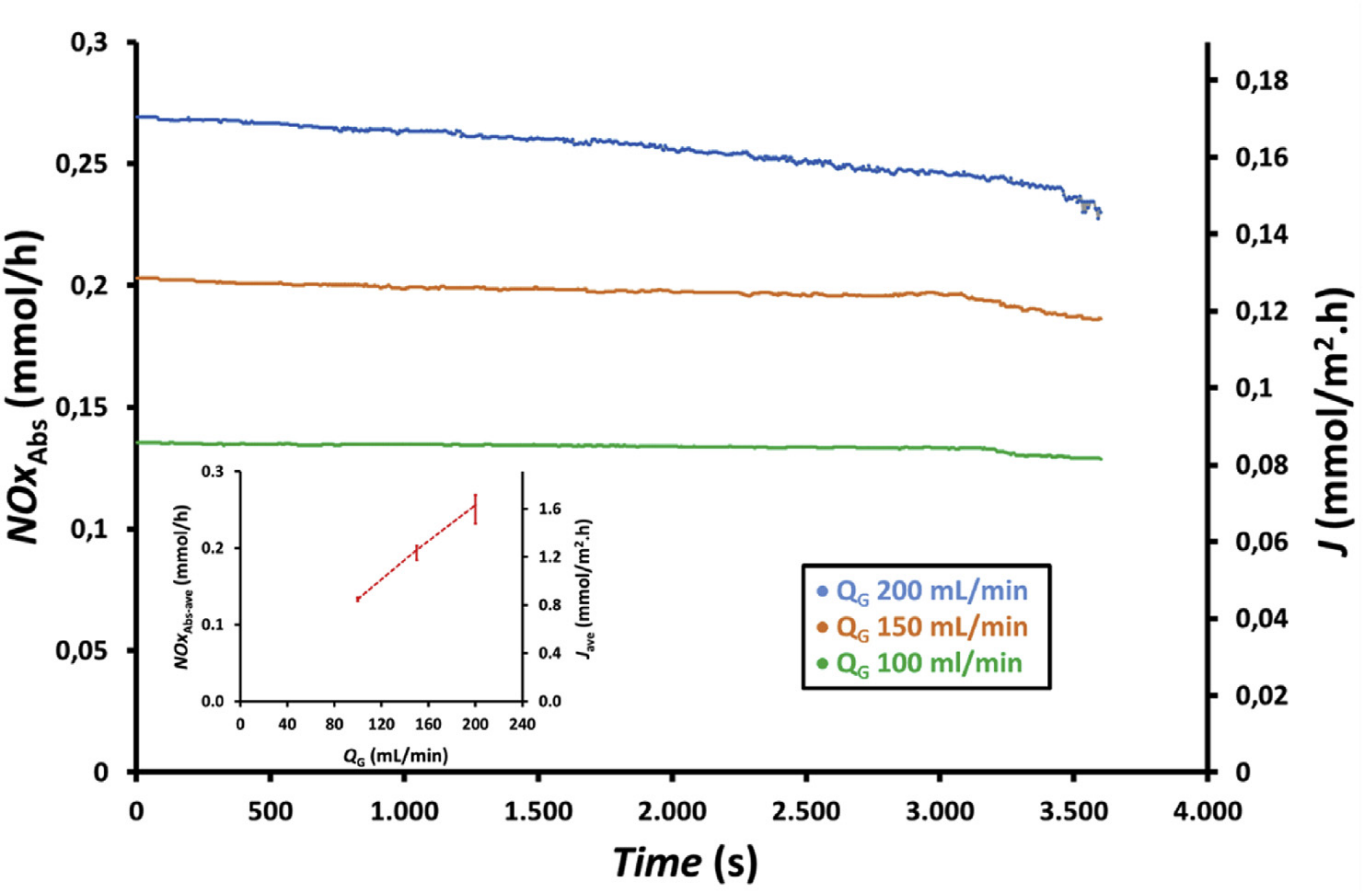
Profiles of the amount of absorbed NOx, *NOx*_Abs_, and flux, *J*, with time at feed gas flow rates, *Q*_G_, of 100, 150, and 200 mL/min and the effect of feed gas flow rates on the averages amount of absorbed NOx, *NOx*_Abs-ave_, and flux, *J*_ave_ (inset figure) using hollow fiber membrane module with 100 fibers and an absorbent solution containing a mixture of 75 mL 0.5 wt.% H_2_O_2_ and 75 mL 0.5 M HNO_3_.

To evaluate the performance of NOx absorption with time, an experiment was conducted using a hollow fiber membrane module with 100 fibers and an absorbent solution containing a mixture of 75 mL of 0.5 wt.% H_2_O_2_ and 75 mL of 0.5 M HNO_3_. Moreover, the feed gas flow rates to the membrane module were 100, 150, and 200 mL/min Figure 4 presents the profile of NOx reduction with time at feed gas flow rates of 100, 150, and 200 mL/min. As shown in the figure, NOx reduction at the feed gas flow rate of 200 mL/min decreased more than that at other two feed gas flow rates. This occurred because absorbed NOx at the gas flow rate of 200 mL/min is also the highest, as shown in Figure 5; thus, the number of moles of the absorbent solution remaining in the shell side of the membrane module also decreased led to the increase NOx concentration in the gas exiting the membrane module, which caused a faster decrease in NOx reduction. In addition, the increasing feed gas flow rate indicated that the gas residence time in the liquid phase in the shell side of the membrane module was increasing. This increase in residence time increased the NOx concentration in the outlet gas of the membrane module, which, according to Eq. (11), caused a decrease in the NOx reduction. During the 1 h experiment, NOx reduction decreased from 0.99 to 0.96, 0.99 to 0.90, and 0.98 to 0.85, i.e., a decrease of approximately 3.0%, 9.1% and 13.3% was achieved for the feed gas flow rates of 100, 150, and 200 mL/min, respectively. Figure 4 (inset) also shows that NOx reduction for an average of 1 h decreases from 0.98 to 0.94 or decreases by approximately 4.1% when the feed gas flow rate increases from 100 to 200 mL/min.

The profiles of absorbed NOx and flux with time at the feed gas flow rates of 100, 150, and 200 mL/min using a hollow fiber membrane module with 100 fibers and an absorbent solution containing a mixture of 75 mL of 0.5 wt.% H_2_O_2_ and 75 mL of 0.5 M HNO_3_ are shown in Figure 5. As shown in Figure 5, absorbed NOx and flux increased with increasing feed gas flow rate due to more NOx in the feed gas that can be absorbed. During the 1hour experiment, absorbed NOx decreased from 0.135 to 0.129 mmol/h, 0.203 to 0.186 mmol/h, and 0.266 to 0.227 mmol/h, i.e., by 4.4%, 8.4%, and 15.6% for the feed gas flow rates of 100, 150, and 200 mL/min, respectively. Therefore, a higher feed gas flow rate caused a higher decrease in NOx absorbed due to the higher amounts of H2O2 and HNO3 consumed during the absorption process, thereby reducing the concentration of the remaining absorbent solution in the shell side of the membrane contactor. Thomas and Vanderschuren [24] reported that NOx reduction during 1 h experiment using water as absorbent decrease from about 60 to about 40%, or a decrease of 33%. Meanwhile, Wang and Yu [25] conducted a longer experiments, which was until 210 min, and found that NOx reduction gradually decreased from about 60 to 45.7%, or a decrease of 23.8%, at feed gas flow rate of 200 mL/min using a mixture of 0.4 wt.% H2O2 and 30 wt.% NaCl as absorbent solution volume of 2 L, absorbent circulation rate of 40 mL/min and temperature of 60 °C.

The profile of flux is identical to that of absorbed NOx as the same membrane surface area was used in the experiments. The NOx absorbed decreased from about 0.135 to 0.131 mmol/h, 0.203 to 0.184 mmol/h and 0.269 to 0.232 mmol/h, or decreases of 3.5, 9.3 and 14.0% when the feed gas flow rates were 100, 150 and 200 mL/min, respectively. Furthermore, the NOx flux, after 1 h experiments, decreased from about 0.86 to 0.83 mmol/m^2^.h, 1.29 to 1.17 mmol/m^2^.h and 1.71 to 1.47 mmol/m^2^.h, or decreases of 3.5, 9.3 and 14.0% when the feed gas flow rates were 100, 150 and 200 mL/min, respectively. Meanwhile, Wang and Yu [25] reported that the NOx flux decreases from 0.065 to 0.0496 mmol/m^2^.h after 210 min of experiment, or a decrease of 23.7%, at feed gas flow rate of 200 mL/min using a mixture of 0.4 wt.% H2O2 and 30 wt.% NaCl as absorbent solution volume of 2 L, absorbent circulation rate of 40 mL/min and temperature of 60 °C. The averages of absorbed NOx and flux during 1 h experiment, as shown in inside Figure 5, increased from 0.133 to 0.255 mmol/h and from 0.85 to 1.63 mmol/m^2^.h or increased about 92% when the feed gas flow rate increased from 100 to 200 mL/min, respectively.

### 3.2 Influence of H_2_O_2_ concentration

**Figure 6:**
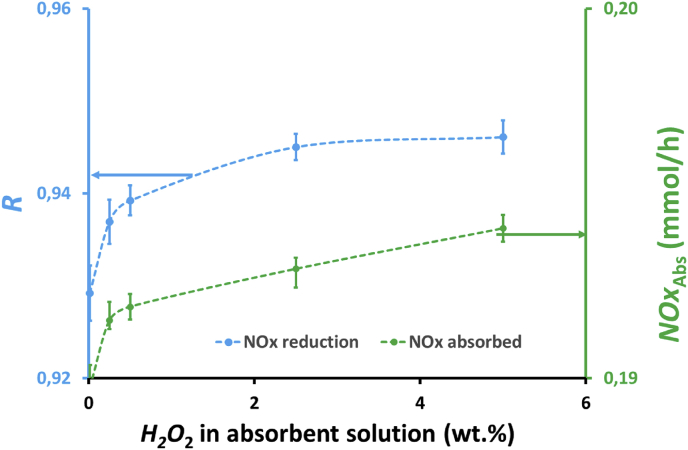
Effect of H_2_O_2_ concentration on NOx reduction, *R*, and the amount of absorbed NOx, *NOx*_Abs_, by the absorbent solution containing 25 mL of H_2_O_2_ and 25 mL of 0.5 M HNO_3_ in a membrane module with 32 fibers and at a feed gas flow rate of 150 mL/min.

**Figure 7:**
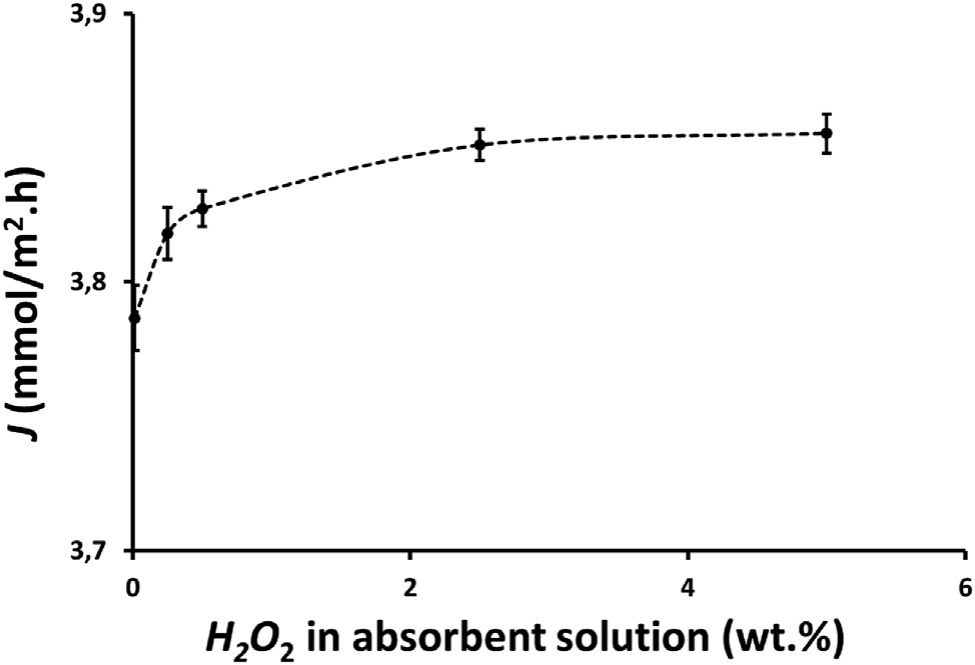
Effect of H_2_O_2_ concentration on the flux, *J*, by the absorbent solution containing 25 mL of H_2_O_2_ and 25 mL of 0.5 M HNO_3_ in a membrane module with 32 fibers and at a feed gas flow rate of 150 mL/min.

The effects of H_2_O_2_ concentration in the absorbent solutions on NOx reduction, *R*, and absorbed NOx, *NOx*_Abs_, are shown in Figure 6. It can be seen that absorbed NOx and NOx reduction increased with increasing H_2_O_2_ concentration in the absorbent solutions because H_2_O_2_ plays an important role in enhancing HNO_2_ oxidation to HNO_3_, as expressed in Eq. (8). NOx reduction and absorbed NOx slightly increased when H_2_O_2_ concentration in absorbent solution was increased from 0.25 to 2.5 wt.%. However, when H_2_O_2_ concentration was increased from 2.5 to 5 wt.%, NOx reduction and absorbed NOx were relatively constant, indicating that at the 2.5 wt.% H_2_O_2_ the maximum NOx absorption was almost achieved. NOx reduction increased from 0.93 to 0.95 when H_2_O_2_ concentration increased from 0.25 to 2.5 wt.%. In addition, a trend similar to that of efficiency occurred in the flux, wherein their values slightly increased when H2O2 concentration increased from 0.25 to 2.5 wt.% and became relatively constant when H2O2 concentration increased from 2.5 to 5 wt.%, as demonstrated in Figure 7. This fact revealed that an increase in H_2_O_2_ concentration from 2.5 to 5 wt.% does not have a significant impact on increasing absorbed NOx as the efficiency of NOx reduction at this range of H_2_O_2_ concentration is already high, as presented in Figure 6. Thus, only a small amount of NOx remaining in the gas phase can be absorbed by the absorbent solution. Wang and Yu [25] reported that there were an increase in NO reduction efficiency from 0.372 to 0.478 when the H_2_O_2_ concentration increased from 0 to 0.4 wt.% at the feed gas flow rate of 200 mL/min containing NO of 184.8 ppm and the absorbent solution of H_2_O_2_ 0 to 0.4 wt.% and NaCl 5 wt.%. In addition, Hao et al. reported that there was an increase in NO reduction from 0.19 to 0.64 and from 0.21 to 0.82 without and with UV light in a hybrid catalytic reactor, respectively, when H_2_O_2_ concentration increased from 1 to 30 wt.% [32]. In addition, Hao et al. also reported that when H_2_O_2_ solution was increased from 50 to 150 μL/min, there was an increase in NO reduction efficiency from 0.416 to 0.625 and from 0.55 to 0.956 without and with UV light, respectively.

**Figure 8:**
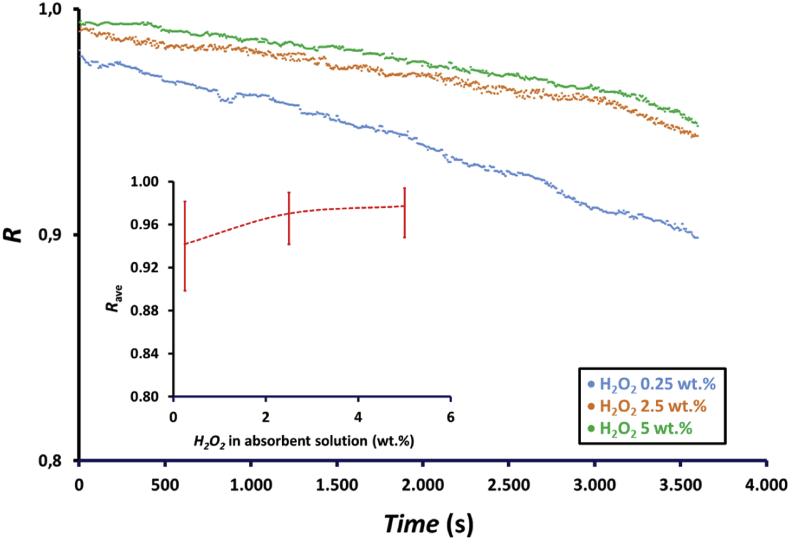
Profile of NOx reduction, *R*, with time and the effect of hydrogen peroxide concentrations on the average NOx reduction, *R*_ave_ (inset figure) at feed gas flow rate, *Q*_G_, of 200 mL/min using a membrane module with 100 fibers and an absorbent solution containing a mixture of 75 mL of 0.5, 5, and 10 wt.% H_2_O_2_ and 75 mL of 0.5 M HNO_3_.

The profile of NOx reduction with time at H_2_O_2_ concentrations of 0.25, 2.5, and 5 wt.% is presented in Figure 8. As shown in Figure 8, the efficiency of NOx reduction, *R*, increased with the increase in the H_2_O_2_ concentration in the absorbent solution because H_2_O_2_ enhances the oxidation of HNO_2_ to HNO_3_, as expressed in Eq. (4). Figure 8 shows the difference in NOx reduction increment when H_2_O_2_ concentration was increased from 0.25 to 2.5 wt.% and from 2.5 to 5 wt.%. There was an increase in NOx reduction when the H_2_O_2_ concentration in the absorbent solution was increased from 0.25 to 2.5 wt.%, whereas NOx reduction only slightly increased when H_2_O_2_ concentration increased from 2.5 to 5 wt.%. Figure 8 also shows that at lower H_2_O_2_ concentrations, the decrease in efficiency is more significant because of low amount of H_2_O_2_ remaining in the absorbent solution with time, which oxidizes HNO_2_ to HNO_3_. During 1 h experiments, NOx reduction decreased from 0.98 to 0.90, 0.99 to 0.95, and 0.99 to 0.94, i.e., decreased by approximately 8.2%, 5.1%, and 4.0% for H_2_O_2_ concentrations of 0.25, 2.5 and 5 wt.%, respectively. Based on the average NOx reduction, *R*_ave_, as shown in the inset of Figure 8, there was in increase from 0.94 to 0.98 or increased by approximately 4.3% when H_2_O_2_ concentration in absorbent solution increased from 0.25 to 5 wt.%.

**Figure 9:**
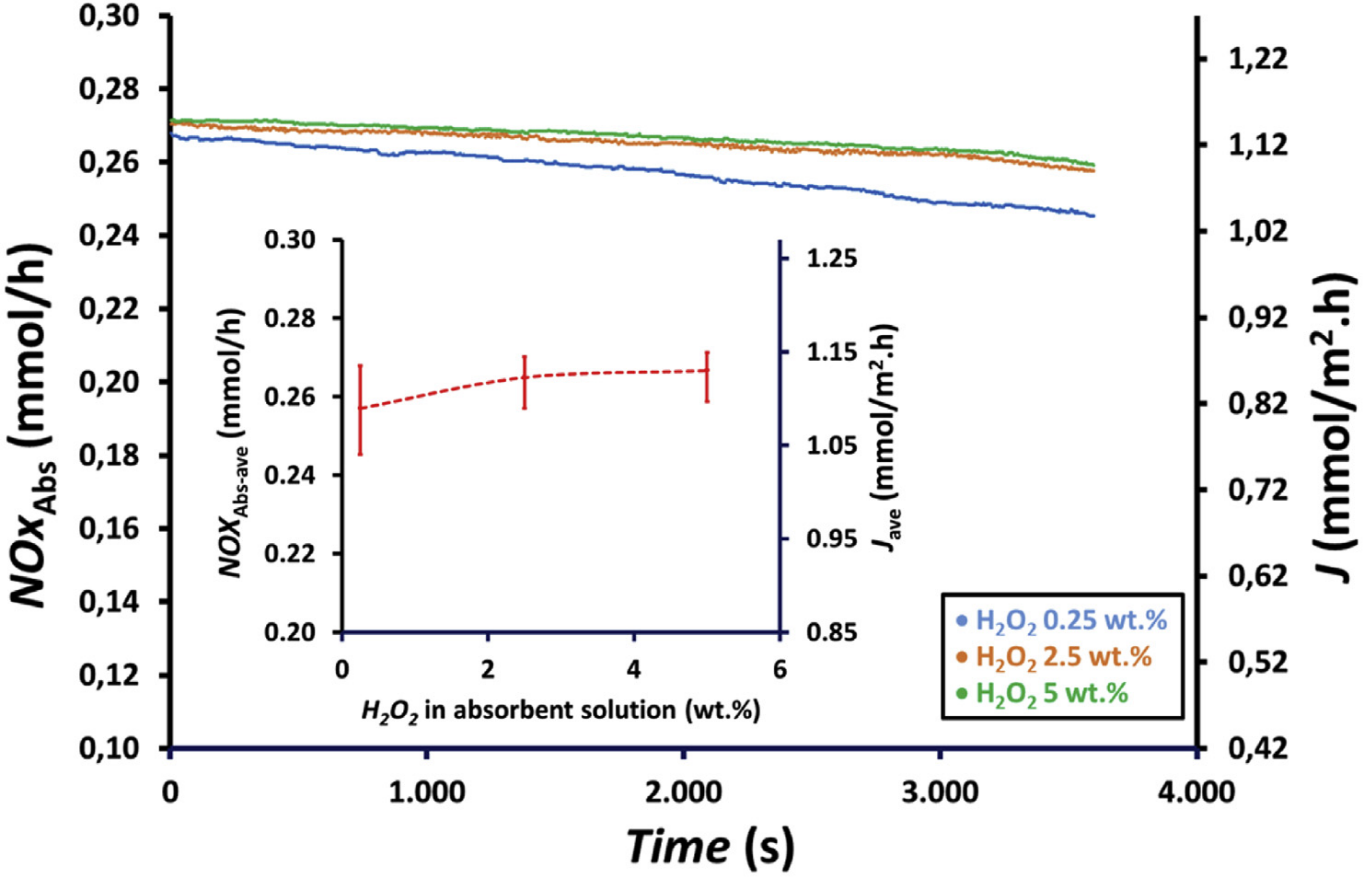
Profile of absorbed NOx, *NOx*_Abs_, and flux, *J*, with time and the effect of hydrogen peroxide concentration on the average amount of absorbed NOx, *NOx*_Abs-ave_ (inset figure) at feed gas flow rate, *Q*_G_, of 200 mL/min using a membrane module with 100 fibers and an absorbent solution containing a mixture of 75 mL of H_2_O_2_ 0.5, 5 and 10 wt.% H_2_O_2_ and 75 mL of 0.5 M HNO_3_.

The effects of H_2_O_2_ concentration on the absorbed NOx, *NOx*_Abs_, and the flux, *J*, with time are presented in Figure 9. Absorbed NOx and flux showed a similar trend because the same membrane module was applied for the experiments. As shown in Figure 9, the absorbed NOx and the flux increased with increasing H_2_O_2_ concentration in the absorbent solution due to the enhance oxidation of HNO_2_ to HNO_3_, as expressed in Eq. (4). Similar to NOx reduction, there was a difference in absorbed NOx increment when H_2_O_2_ concentration was increased from 0.25 to 2.5 wt.% and from 2.5 to 5 wt.%. There was also a more significant decrease in NOx absorption at lower H_2_O_2_ concentrations. The absorbed NOx increased when H_2_O_2_ concentration increased from 0.25 to 2.5 wt.%, whereas absorbed NOx only slightly increased when H_2_O_2_ concentration increased from 2.5 to 5 wt.%. During 1 h experiments, the absorbed NOx decreased from 0.268 to 0.245, 0.270 to 0.257, and 0.271 to 0.259, i.e., decreased by 8.5%, 4.9%, and 4.6% for H_2_O_2_ concentrations of 0.25, 2.5 and 5 wt.%, respectively. The average absorbed NOx and flux in the 1hour experiment are shown in the inset of Figure 9. The average absorbed NOx and flux increased from 0.258 to 0.267 mmol/h and from 1.09 to 1.13 mmol/m^2^.h, respectively, when H_2_O_2_ concentration was increased from 0.25 to 5 wt.%. There was an increase by approximately 2.8% in absorbed NOx and flux when H_2_O_2_ concentration increased from 0.25 to 2.5 wt.%, whereas absorbed NOx and flux only increased by approximately 0.7% when H_2_O_2_ concentration increased from 2.5 to 5 wt.%. This result revealed that an increase in H_2_O_2_ concentration from 2.5 to 5 wt.% does not have a significant impact on absorbed NOx and flux as the efficiency of NOx reduction at this concentration range is already high, as presented in Figure 8. Therefore, only a small amount of NOx remaining in the gas phase can be absorbed by the absorbent solution.

### 3.3 Influence of fiber number in the hollow fiber membrane module

**Figure 10:**
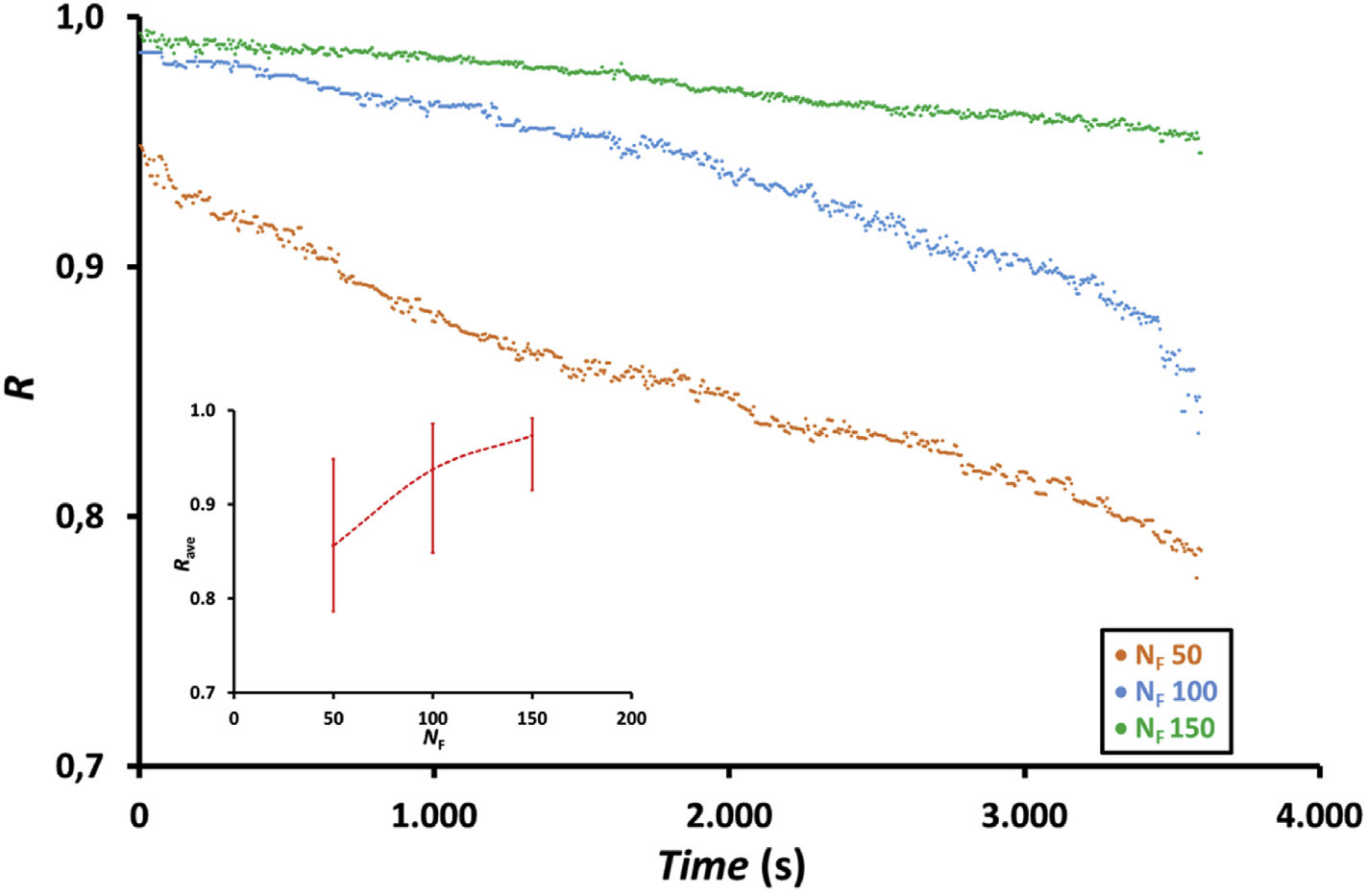
Profile of NOx reduction, *R*, with time and the effect of the number of fibers in the membrane module, *N*_F_, on the average NOx reduction, *R*_ave_ (inset figure) at feed gas flow rate, *Q*_G_, of 200 mL/min using a membrane module with 50, 100 and 150 fibers and absorbent solution containing a mixture of 75 mL of 0.5 wt.% H_2_O_2_ and 75 mL of 0.5 M HNO_3_.

The effect of fiber number in the membrane modules on NOx reduction at a feed gas flow rate of 200 mL/min using an absorbent solution containing a mixture of 75 mL of H_2_O_2_ 0.5 wt.% and 75 mL of 0.5 M HNO_3_ is presented in Figure 10. The NOx reduction increased with increasing number of fibers in the membrane module. At the same feed gas flow rate, the amount of gas flowing over a single fiber in the membrane module decreases with increasing number of fibers in the membrane module, which is unfavorable for NOx absorption as shown in Figure 2. However, the gas released by a single membrane has a longer residence time compared to the gas released by a single fiber in the module membrane that have fewer fibers; thus, increasing fiber numbers enhances the NOx absorption process. Longer the gas residence time and larger the surface area for gas-liquid contact, better was the gas–liquid contact in the shell side of the membrane module, which is favorable for the NOx absorption. As NOx reduction increased with increasing the number of fibers in the membrane module, it reveals that the effects of residence time and surface area for gas–liquid contact were more dominant than that of the feed gas flow rate in a single fiber. The average NOx reduction, as presented in the inset of Figure 10, increased from 0.86 to 0.97, i.e., increased by approximately 12.8% when the number of fibers in the membrane modules increased from 50 to 150, respectively. The enhanced NOx reduction results have also been reported by Yang et al. [33] and Cui et al. [34]. Yang et al. reported that NOx removal can achieve 80% efficiency when 2 M H2O2 was used as the absorbent solution at a flow rate of 0.007 mL/min in a catalytic reactor using alkali-magnetically modified fly ash catalyst, reaction temperature of 137 °C, feed gas flow rate of 300 mL/min, and NOx concentration of 350 ppm [33]. Moreover, Cui et al. reported that 90% NOx removal efficiency was achieved at 140 °C, H2O2 flow rate of 0.07 mg/min, and 250 mL/min gas stream containing 500 ppm NO in a fix bed reactor using a catalyst derived from alkali-acid modification [34].

**Figure 11:**
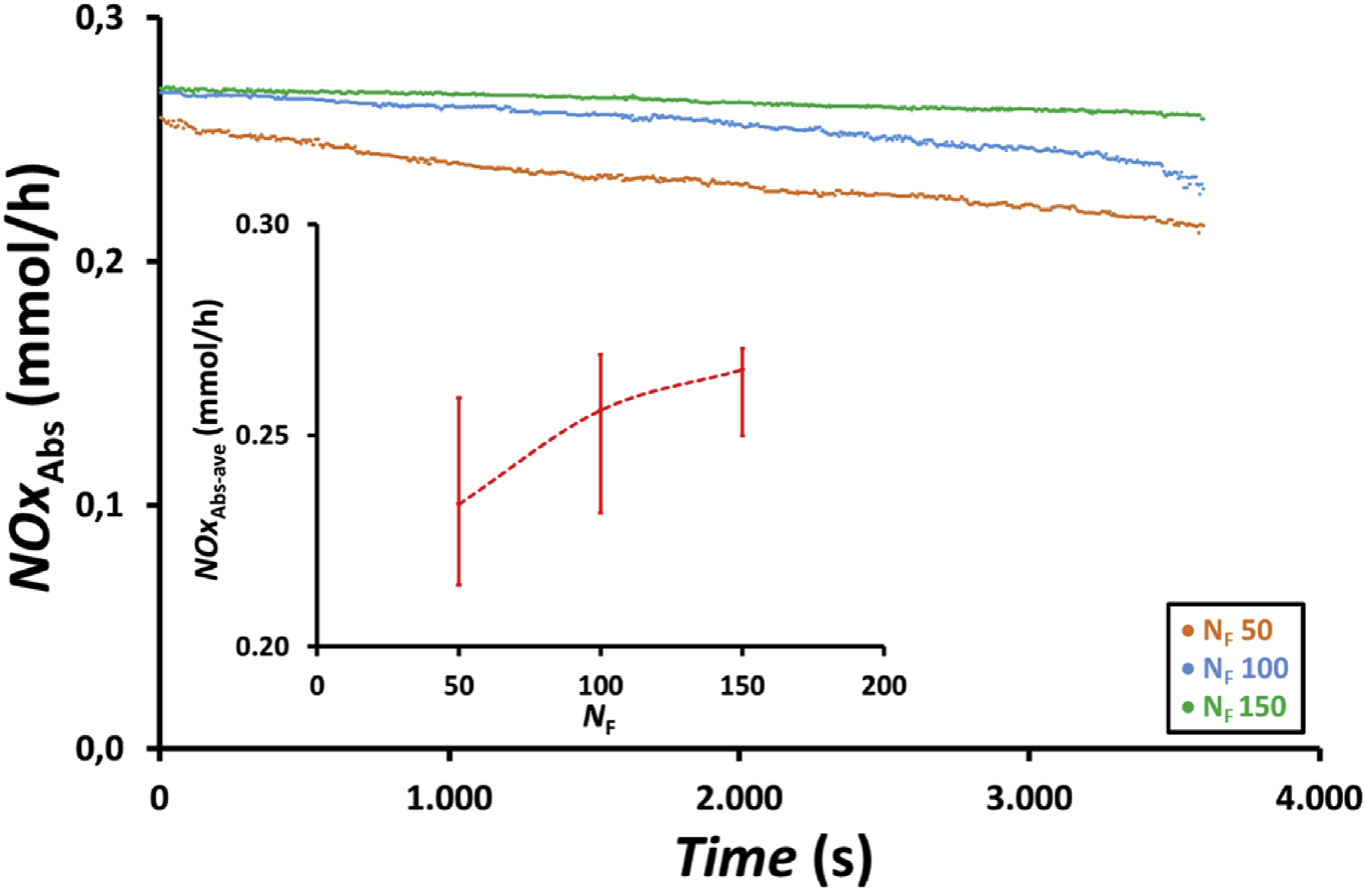
Profile of absorbed NOx, *NOx*_Abs_, with time and the effect of the number of fibers in the membrane module, *N*_F_, on the average amount of absorbed NOx, *NOx*_Abs-ave_ (inset figure) at feed gas flow rate, *Q*_G_, of 200 mL/min using membrane module with 50, 100, and 150 fibers and an absorbent solution containing a mixture of 75 mL of 0.5 wt.% H_2_O_2_ and 75 mL of 0.5 M HNO_3_.

The effect of fiber number in the membrane module on absorbed NOx, *NOx*_Abs,_ at a feed gas flow rate of 200 mL/min using an absorbent solution containing a mixture of 75 mL of 0.5 wt.% H_2_O_2_ and 75 mL of 0.5 M HNO_3_ is presented in Figure 11. Similar to NOx reduction, absorbed NOx increased with increasing number of fibers in the membrane module because of the increased residence time and large surface area for gas–liquid contact. The average absorbed NOx as shown in the inset of Figure 11, increased from 0.23 to 0.27 mmol/h, i.e., increased by 17.4%, when the number of fibers in the membrane module increased from 50 to 150.

**Figure 12:**
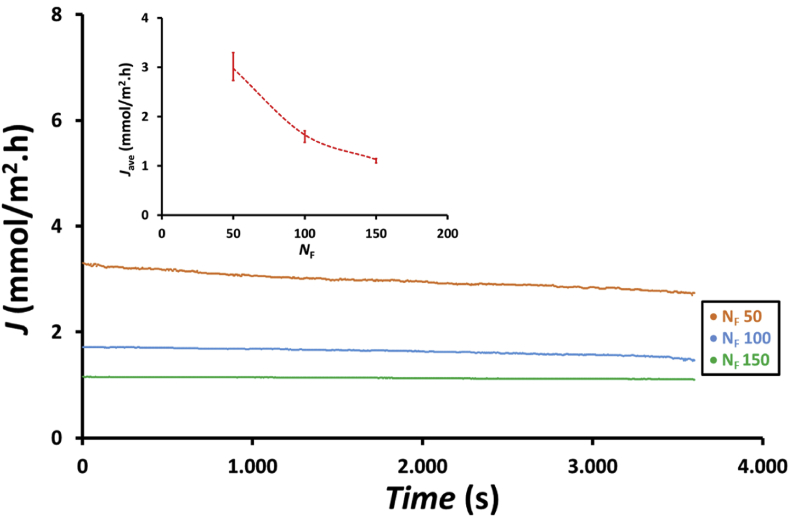
Profile of flux, *J*, with time and the effect of the number of fibers in the membrane module, *N*_F_, on the average flux, *J*_ave_ (inset figure) at feed gas flow rate, *Q*_G_, of 200 mL/min using membrane module with 50, 100 and 150 fibers and an adsorbent solution containing a mixture of 75 mL of H_2_O_2_ 0.5 wt.% and 75 mL of 0.5 M HNO_3_.

Because the number of fibers in the membrane module is different, the flux shows a trend different from that of *NOx*_Abs_. As shown in Figure 12, the flux decreased with the increase in the number of fibers in the membrane module. Increasing the number of fibers in the membrane module increased absorbed NOx, as shown in Figure 11; therefore, it can increase flux, as expressed in Eq. (11). However, increasing the number of fibers also increased the surface area for the gas–liquid contact; thus, according to Eq. (6), this increase reduced the flux. The decrease in flux due to increasing the number of fibers in the membrane module indicated that the effect of surface area for the gas–liquid contact is more dominant than the effect of absorbed NOx. The average flux during the 1hour experiment is shown in the inset of Figure 12. As can be seen, the average flux decreased from 2.98 to 1.13 mmol/m^2^.h, i.e., decreased by approximately 62%, when the number of fibers in the membrane module was increased from 50 to 150.

## 4 Conclusion

In conclusion, based on the major findings of this study, it can be elicited that the hollow fiber membrane module can be used to reduce NOx. This is realized from the gas stream where the fibers serve to distribute the feed gas prior to having a contact with the absorbent solution in the shell side side of the membrane module. This results in an increase in the surface area for gas-liquid contact. This increment is very beneficial for the reaction between NOx in the gas stream and the absorbent solution containing hydrogen peroxide and nitric acid in the shell side of membrane module. This study observed the effects of feed gas flow rate, hydrogen peroxide concentration and the number of fibers in the membrane module on the NO_x_ reduction, as well as NOx absorbed and flux. The experimental results showed that an increase in the feed gas flow rate from 100 to 200 mL/min resulted in a decrease in NOx reduction from 98 to 94% but significantly effected an increase in the absorbed NOx and flux from about 0.13 to 0.26 mmol/h and 0.85–1.63 mmol/m^2^.h, respectively, due to the fact that an increase in NO_x_ concentration in the feed gas effect was dominant than an increase in absorbed NOx. An increase in hydrogen peroxide concentration from 0.25 to 5 wt.% in the absorbent solutions increased NOx reduction, absorbed NOx and flux from 94 to 98%, 0.257–0.267 mmol/h and 1.09–1.13 mmol/m^2^.h. This resulted to increment approximately 4.3, 3.5 and 3.7%, respectively as H_2_O_2_ plays an important role in enhancing HNO_2_ oxidation to HNO_3_. Furthermore, an increase in the number of fibers from 50 to 150 in the membrane module increased NOx reduction and absorbed NOx from about 86 to 97% and 0.23–0.27 mmol/h. The percentage increment is approximately 12.8 and 17.4, respectively. The flux decreased from 2.98 to 1.13 mmol/m^2^.h, representing an approximate of 62% decreased due to an increase in gas-liquid contact surface area. Subsequently, the performance of other applicable absorbent solutions for NOx reduction should be addressed to find the best solution for NOx reduction through the hollow fiber membrane module.

## Declarations

### Author contribution statement

Sutrasno Kartohardjono: Conceived and designed the experiments; Performed the experiments; Analyzed and interpreted the data; Contributed reagents, materials, analysis tools or data; Wrote the paper.

Clarissa Merry, Mohamad Sofwan Rizky & Catharina Candra Pratita: Performed the experiments; Analyzed and interpreted the data; Contributed reagents, materials, analysis tools or data.

### Funding statement

This work was supported by the Q1Q2 Project of Universitas Indonesia through Grant No. NKB-0326/UN2.R3.1/HKP.05.00/2019.

### Competing interest statement

The authors declare no conflict of interest.

### Additional information

No additional information is available for this paper.
